# Modeling the Transmission of *Vibrio aestuarianus* in Pacific Oysters Using Experimental Infection Data

**DOI:** 10.3389/fvets.2019.00142

**Published:** 2019-05-14

**Authors:** Coralie Lupo, Marie-Agnès Travers, Delphine Tourbiez, Clément Félix Barthélémy, Gaël Beaunée, Pauline Ezanno

**Affiliations:** ^1^Laboratoire de Génétique et Pathologie des Mollusques Marins, SG2M-LGPMM, Ifremer, La Tremblade, France; ^2^BIOEPAR, INRA, Oniris, Nantes, France

**Keywords:** marine epidemiology, parameter estimation, compartmental model, ABC method, global sensitivity analysis, basic reproduction number R_0_, *Crassostrea gigas*, oyster mortality

## Abstract

*Vibrio aestuarianus* is a bacterium related to mortality outbreaks in Pacific oysters, *Crassostrea gigas*, in France, Ireland, and Scotland since 2011. Knowledge about its transmission dynamics is still lacking, impairing guidance to prevent and control the related disease spread. Mathematical modeling is a relevant approach to better understand the determinants of a disease and predict its dynamics in imperfectly observed pathosystems. We developed here the first marine epidemiological model to estimate the key parameters of *V. aestuarianus* infection at a local scale in a small and closed oyster population under controlled laboratory conditions. Using a compartmental model accounting for free-living bacteria in seawater, we predicted the infection dynamics using dedicated and model-driven collected laboratory experimental transmission data. We estimated parameters and showed that waterborne transmission of *V. aestuarianus* is possible under experimental conditions, with a basic reproduction number R_0_ of 2.88 (95% CI: 1.86; 3.35), and a generation time of 5.5 days. Our results highlighted a bacterial dose–dependent transmission of vibriosis at local scale. Global sensitivity analyses indicated that the bacteria shedding rate, the concentration of bacteria in seawater that yields a 50% chance of catching the infection, and the initial bacterial exposure dose W_0_ were three critical parameters explaining most of the variation in the selected model outputs related to disease spread, i.e., R_0_, the maximum prevalence, oyster survival curve, and bacteria concentration in seawater. Prevention and control should target the exposure of oysters to bacterial concentration in seawater. This combined laboratory–modeling approach enabled us to maximize the use of information obtained through experiments. The identified key epidemiological parameters should be better refined by further dedicated laboratory experiments. These results revealed the importance of multidisciplinary approaches to gain consistent insights into the marine epidemiology of oyster diseases.

## Introduction

Mass mortality of adult Pacific oysters, *Crassostrea gigas*, has been reported since 2001 in France, in association with the detection of the bacterium *Vibrio aestuarianus*. Since 2011, an increase in the incidence of these mortality events has been observed (1). This bacterium has been isolated recently during oyster mortality events also in Ireland and Scotland (2, 3). Such an increase in mortality has a strong direct economic impact, causing considerable concerns among farmers because adult oysters that have reached their marketable size are mainly affected (4). Mortality mainly occurs in summer and seems to last for a long period, reaching a cumulative mortality rate of ~30% at the end of the farming cycle (1). To date, knowledge about this infection mainly concerns the properties of the etiological agent *V. aestuarianus*, its diversity (5, 6) and virulence factors (5, 7), the diagnostic methods available (8), and the potential genetic basis for oyster resistance to the induced disease (4, 9). The burden of *V. aestuarianus* infection in oysters has been less studied. Hemolymph is colonized early by the bacterium, and the disease rapidly progresses through septicemia (10). Experimental reproduction of the disease in cohabitation trials has shown that oyster sensitivity increases with age and size, mortality rates reaching up to 75% during the 15 days post-exposure (dpe) (4, 11). Oyster mortality rates increase with the bacterial concentration in seawater, indicating a dose-dependent process in disease progression (12). Because DNA of *V. aestuarianus* has not been detected in surviving challenged oysters (12), it seems that these oysters never got infected. This observation also suggests that the oyster once infected never returns to a truly uninfected state, death being the sole outcome. The infection kinetics seems to be modulated by seawater temperature: the warmer the water, the faster is the infection (13), infection occurring within a range from 9–13°C to 19–20°C (3). Attention has naturally focused on the biology of the individual organism. To date, knowledge about pathogen transmission has been ignored. As a result, facing outbreaks has typically given rise to mainly observation of cases and their possible drivers rather than the implementation of efficient control measures. The quantification of the transmission of these bacteria is a worthwhile step for further elucidating the disease spread and establishment in oyster populations, and to substantially inform disease management.

Mathematical modeling can be used to provide new insights on the relative importance of factors influencing disease spread (14). Such an approach synthesizes knowledge about a disease into a quantitative framework (15). The choice of an appropriate mode of a transmission model is also crucial for designing a proper intervention strategy (16). To date, despite the acknowledged application of susceptible-infectious-recovered (SIR) compartmental modeling framework to marine diseases (16, 17) and to their invertebrate hosts (18), epidemiological models of pathogen transmission remain on the fringe of marine disease modeling, partly because of limited amounts of data (19, 20). Examples of disease dynamics among marine invertebrates have previously been formulated, e.g., describing direct waterborne transmission of white plague type in corals (21, 22) or withering syndrome in abalone (23). Bivalve diseases have received less attention. The possible transmission mechanisms of bonamiosis in flat oyster *Ostrea edulis* populations have been explored theoretically by comparing five compartmental models, without relating them to any particular case study (24). More recently, a theoretical compartmental model was formulated to represent the transmission of diseases among marine suspension-feeders (25), e.g., oysters. The mode of transmission was assumed to involve contacts between the host and the free-living stages of the pathogen in seawater, described there by filtration of infective particles released by infected and/or dead individuals, including dose dependence and dilution via volume of the water column (25). This transmission model explicitly accounts for pathogen population multiplication or reduction inside and outside its host and includes several explicit compartments for waterborne pathogens, a filtered pool of pathogens in hosts, and a remote pool of pathogens (25, 26). This conceptual model was further adapted to simulate the dynamics of *Perkinsus marinus*, a well-known parasite of the Eastern oyster *Crassostrea virginica* (27), via the incorporation of seasonal factors. However, there is currently no knowledge about the *V. aestuarianus* environmental populations, thus preventing the formulation of such a detailed model. Besides, as this bacterium belongs to the genus *Vibrio*, a direct waterborne transmission among oysters similar to *Vibrio cholerae* can be assumed. Mathematical modeling of cholera transmission among humans, where the causative agent is the bacterium *V. cholerae*, has become exemplary within the framework of environmentally transmitted infectious diseases (28). Cholera models are mainly formulated via SIR compartmental modeling framework and imply that individuals become infected via the consumption of contaminated seawater, therefore including an explicit compartment for the aquatic reservoir of pathogens (29). This model formulation could thus be adapted to *V. aestuarianus*, considering an explicit compartment for free-living bacteria in seawater but without detailing all of the pathogen multiplication processes.

Except for one (27), the parameterization of existing marine invertebrate disease models has been based on theoretical parameter values. These have been chosen arbitrarily, albeit biologically plausible (21–26). In any host–pathogen system, epidemiological parameters are difficult to measure directly (30). Indeed, because diseased mollusks show only very seldom symptoms, mortality event is often the only suspicious sign of disease observed in the field, and laboratory test analyses are necessary to identify the causative pathogen (31). In France, the current surveillance system is mainly based on the notifications of mollusk mortality by farmers, and laboratory investigation of these notifications is not systematic (32). In addition, as *V. aestuarianus* is not a notifiable disease, no active surveillance of this pathogen is conducted. Consequently, field data are insufficient to quantify the transmission of marine molluscan diseases.

To overcome this lack of observation data, the quantification of epidemiological parameters can be performed experimentally (30). Several studies have attempted to quantify these parameters under experimental conditions to further inform mathematical models of marine infectious diseases (33, 34). Some small-scale laboratory experiments have been carried out to explore bacterial shedding, the minimum infective dose, and half-lethal dose (LD_50_) of *V. aestuarianus* infection among oysters (12). Nevertheless, parameters have been estimated independently in separate experiments, thereby probably leading to misestimating their value because the assumption of independence among parameters may be violated. In addition, due to logistical constraints, only small populations can be studied under experimental conditions. As these parameters most probably are highly heterogeneous among individuals, it is difficult to accurately estimate them, requiring population scale experiments.

Thus, given that the quantification of epidemiological parameters is not straightforward, it becomes crucial to first identify the key parameters whose variations strongly influence model outputs, in order to subsequently focus research investment for reducing model prediction uncertainty. Key parameters can be identified by analyzing the model sensitivity, i.e., by studying the effects on model outputs of varying input epidemiological parameters.

Our objective was to better understand and predict *V. aestuarianus* transmission in Pacific oysters using experimental data. We developed a mathematical model based on the model for *V. cholera* (29) and fitted to data on *V. aestuarianus* from dedicated laboratory experiments. Using the model, we (1) reproduced the transmission of *V. aestuarianus* among oysters (2), evaluated the ability of the pathogen to invade an oyster population under experimental small-scale conditions reproducing natural course of the infection (3). identified epidemiological parameters influencing pathogen spread the most within a small oyster population, and (4) provided probable range estimates of these parameters.

## Materials and Methods

### Description of the Natural System

*V. aestuarianus* infection is transmitted through a direct waterborne process (10). Infected oysters shed bacteria in water until their death. When exposed to contaminated water, new oysters can get infected. After an incubation period during which the bacteria invade the oyster, infected oysters start shedding bacteria in the seawater.

### Experimental Design

#### Animals

Batches of Pacific oyster *C. gigas* were produced in March 2013 in Ifremer Argenton (Bretagne, France), transferred in May to the Ifremer nursery at Bouin facilities (Vendée, France) and in November 2014 to the Ifremer experimental facilities in La Tremblade (Charente-Maritime, France). The oysters were housed in 240 L raceways with a continuous ultraviolet (UV) light–treated seawater flow and an *ad libitum* phytoplankton diet (*Isochrysis galbana, Tetraselmis suecica*, and *Skeletonema costatum*). Oysters used in the experiments in September 2015 weighted by mean 12.1 g ± 1.6 (body weight). Twelve oysters were screened before experiments for *V. aestuarianus* and OsHV-1 herpes virus DNA by standardized quantitative PCR (qPCR) protocols (8, 35) to ensure that the batches were not infected before the transmission trials. This sample size calculation was based on the assumption that both infections would exist in the population above a prevalence of 20–30% in permissive conditions, i.e., allowing their progression (10).

#### Bacterial Strains and Inoculum Preparation

The GFP-tagged *V. aestuarianus* strain used in this study was derived from wild-type strain 02/041 (36, 37). *Vibrio* isolates were grown in the Luria-Bertani (LB) medium or LB agar supplemented with 0.5 M NaCl in the presence of kanamycin (40 μg/mL), at 20°C. The cells were washed twice with filtered sterile seawater before adjustment to optical density at 600 nm (OD_600_) of 1.0. Bacterial concentration and purity were checked by conventional dilution plating.

#### Contaminated-Seawater Preparation

Oysters were myorelaxed for 2–3 h at 20°C in a magnesium chloride solution (MgCl_2_, Sigma-Aldrich) at a concentration of 50 g/L (1/4 [v/v] seawater/freshwater) with aeration. Next, 100 μL of a bacterial suspension was injected into the adductor muscle. A group of 10 oysters was injected with filtered sterile seawater as negative controls. The inoculated oysters were then transferred to tanks (60 oysters per tank) filled with 12 L of UV light–treated and filtered seawater and maintained at 20°C under static conditions with aeration. After 48 h, the contaminated seawater surrounding moribund oysters was titrated by flow cytometry and qPCR before adjustment to two doses (low and high doses; between 5 × 10^4^ and 1 × 10^6^ bacteria/mL) by dilution in UV light–treated and filtered seawater.

#### Transmission Trials

**Figure 1:**
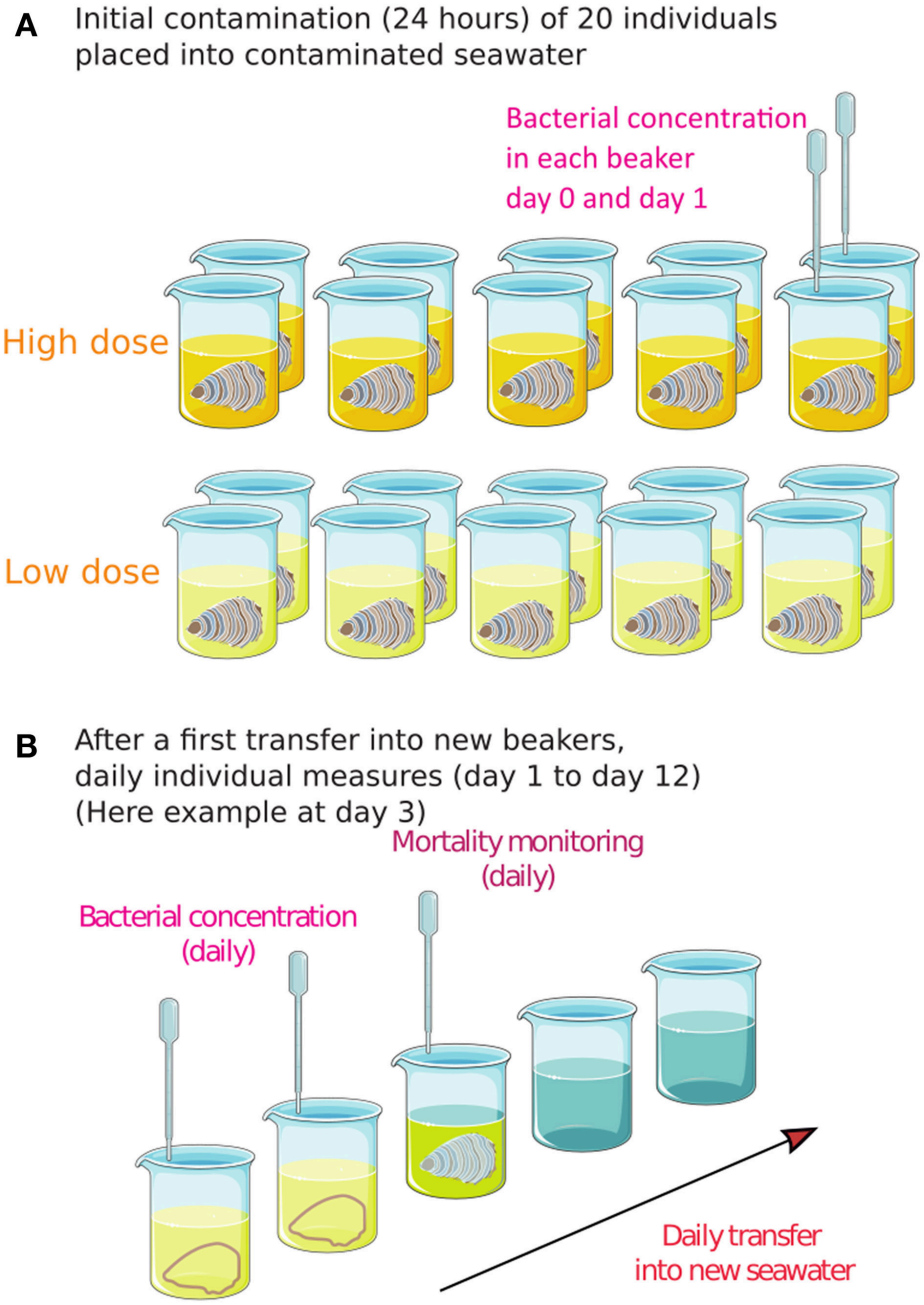
The experimental design for assessing *Vibrio aestuarianus* transmission from contaminated seawater to contact oysters. **(A)** Initial exposure of 10 oysters × 2 doses for 24 h. **(B)** After transfer of each individual oyster into a new beaker with clean seawater, daily individual measures were conducted for 12 days. As soon as bacteria were detected in the seawater, the oyster was transferred into a new beaker, and the survival of bacteria was measured daily in the previous beaker (now empty).

Oysters (2 dose conditions × 10 oysters) were transiently exposed to the contaminated seawater by immersion in individual aerated containers (300 mL) for 24 h at 20°C before transfer into clean beakers. All the containers were monitored daily by collection of seawater samples. As soon as *V. aestuarianus* was detected in seawater by flow cytometry, oysters in contact with contaminated seawater (hereafter: contact oysters) were assumed to be infectious, were removed from the container, and daily transferred into new 300 mL containers with UV light–treated and filtered seawater. Containers with shed bacteria were followed for up to 7 days after oyster removal, by flow cytometry and qPCR. Contact oyster mortality was also recorded daily for 12 dpe, and dead oysters were daily removed. The animals were considered dead when the valves did not close after transfer on tank covers. Infection by *V. aestuarianus* was confirmed by qPCR in oyster tissue samples. Figure 1 summarizes the experimental design.

Three temporal individual trials were conducted (2 dose conditions × 3 trials × 10 oysters) in this study. Moreover, in two of these trials, complementary experiments and measures were conducted on small populations of 10 oysters in triplicate in 3 L tanks (2 dose conditions × 2 trials × 3 populations of 10 oysters), allowing 12 replicates of the transmission trials at a small population level.

#### V. Aestuarianus Counts and Detection

The presence of GFP-tagged bacteria and bacterial counts were measured in 100 μl of sampled seawater by flow cytometry (Coulter Epics XL cytometer, Beckman®, and CyFlow Cube 6 Robby Partec®) on 10,000 events or after 5 min with a fixed threshold of FL1 fluorescence. This method enabled measurement of a concentration of *V. aestuarianus* ≥10^3^ bacteria/mL. *V. aestuarianus* DNA amounts were measured by qPCR detection methods (8) in oyster tissues (50 mg) or seawater (100 μL) after DNA extraction via QiaAmp® tissue kit procedures (Qiagen®) and total-DNA adjustment to 5 ng/μL when needed. A standard curve was built by means of serially diluted titrated genomic extracts (12). Assays were performed on MX3000 and MX3005 machines (Agilent®) with the Brilliant III Ultrafast kit (Stratagene®).

### Modeling

Based on a study on a *Vibrio* bacterium (29) and on new experimental data (this study), a stochastic compartmental model of the SWEI (susceptible-water-exposed-infectious) type was chosen to represent *V. aestuarianus* transmission among oysters in a small-scale population (§2.3.1). In this system, infection transmission occurred through contacts between susceptible oysters and the contaminated seawater, i.e., through direct waterborne transmission. Parameters were calibrated using an Approximated Bayesian Computation (ABC) approach (§2.3.2) using the new experimental data described above.

#### Model Formulation

By being exposed to contaminated water (W) containing bacteria, a susceptible oyster (*S*) became infected (E) at rate *a*.λ(W), where *a* is the rate of effective exposure to the bacteria in the contaminated water (per day) and λ(W) is the probability (unitless) of a susceptible oyster to catch the infection. An infected oyster became infectious (I), i.e., started shedding an amount of bacteria per mL and per day (e), after a latent period (1/ρ), and shed during an infectious period (1/*r*). After initiation of the infection, we assumed that the only outcome for an oyster was death (9, 12). To reproduce the lab experiment in which dead oysters were removed every day, no state was considered in the model for dead animals. Therefore, each oyster was in one of the three mutually exclusive health states at a given time point: S (susceptible oyster), E (non-shedder infected oyster), or I (shedder infectious oyster). Let N denote the total alive population size, i.e., N = S + E + I. Shedders (I) filled the water compartment (W) with bacteria. The decay rate of *V. aestuarianus* in the seawater ξ included the natural mortality, sedimentation, and other stages of the bacterium that prevent its transmission to susceptible oysters. In absence of evidence of *V. aestuarianus* persistence in the seawater (10, 38, 39), the growth rate of the bacterium in this compartment was neglected.

**Figure 2:**
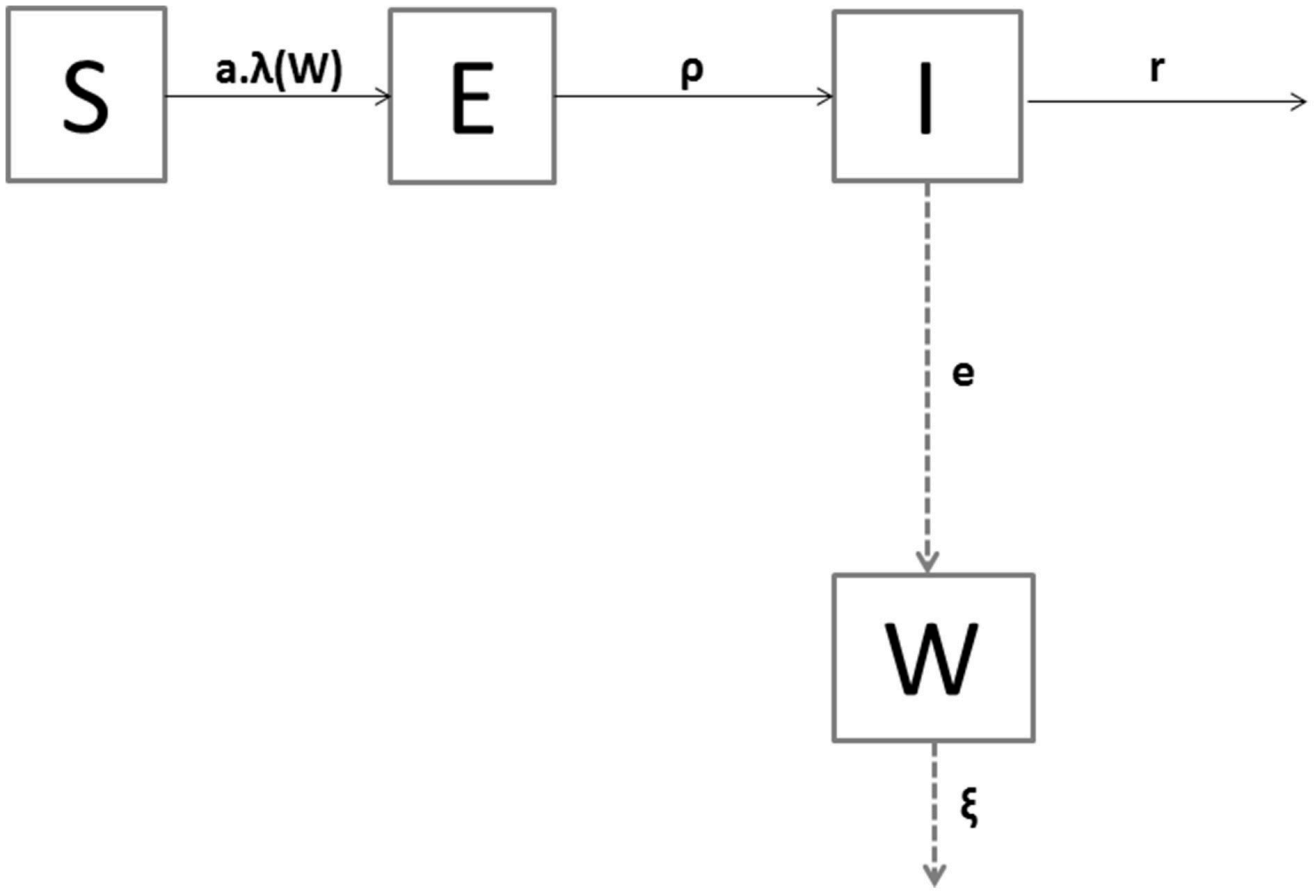
Compartmental model of *Vibrio aestuarianus* spread among oysters and in their environment. The health states of oysters are S for susceptible, E for exposed, and I for infectious. W denotes the seawater concentration of free-living bacteria. Solid arrows indicate oyster transitions between heath states. Dashed arrows represent the dynamics of the pathogen population. The description of parameters is the same is in Table 1.

A flow diagram of the model is shown in Figure 2, and parameters are defined in Table 1. The temporal dynamics of *V. aestuarianus* in the oyster population and in seawater was described by the following system of ordinary differential equations [equations (1) to (4)] in a deterministic framework:

(1) $$\begin{array}{lll} \frac{\text{dS}}{\text{dt}} & = & -a\cdot \lambda \left(W\right)\cdot S \end{array}$$

(2) $$\begin{array}{lll} \frac{\text{dE}}{\text{dt}} & = & a\cdot \lambda \left(W\right)\cdot S-\rho \cdot E \end{array}$$

(3) $$\begin{array}{lll} \frac{\text{dI}}{\text{dt}} & = & \rho \cdot E-r\cdot I \end{array}$$

(4) $$\begin{array}{lll} \frac{\text{dW}}{\text{dt}} & = & e\cdot I-\xi \cdot W \end{array}$$

Probability of catching *V. aestuarianus* infection λ(W) was extrapolated from studies on *V. cholera* (29) and depended on the concentration of the pathogen in water. This dependence was represented by a logistic dose–response function as:

(5) $$\begin{array}{l} \lambda \left(W\right)=\frac{W}{K+W}, \end{array}$$

where *K* is the concentration of bacteria in water that yields a 50% chance of catching infection, i.e., the half-infective dose.

**Table 1:** Definition of model parameters.

**Notation**	**Definition (unit)**
*a*	Rate of exposure to contaminated water (days^−1^)
*K*	Concentration of bacteria in water that yields a 50% chance of catching the infection in one day or the half-infective dose (bacteria/mL)
1/ξ	Free-living–bacteria lifetime in seawater (days)
1/ρ	Latency period (days)
*e*	Bacteria shedding rate (bacteria/mL per day per oyster)
1/*r*	Infectious period (days)
λ(W)	Probability of catching *V. aestuarianus* infection, depending on the bacterial concentration in the water and on the parameter K

Because the population was small, a stochastic counterpart of the ordinary differential equation system was run in discrete time. Health transitions and the bacteria decay rate (τ_*ij*_) were transformed into probabilities (*p*_*ij*_) as follows: for each transition from compartment *i* to compartment *j, p*_*ij*_ = 1–exp(–Δ*t*τ_*ij*_). The flow of individuals between compartments *i* and *j* (Δ*N*_*ij*_) was then Δ*N*_*ij*_ = Binomial (*N*_*ij*_, *p*_*ij*_), with *N*_*ij*_ being the number of individuals in compartment *i*. The bacteria shedding rate followed a Poisson distribution. The model was solved using a daily time step, with a closed population. The behavior of the model was analyzed by running 10,000 numerical simulations.

#### Model Parameterization and Initial Conditions

The model parameters were calibrated by integrating knowledge from experimental individual trials.

Because we assumed that after initiation of *V. aestuarianus* infection, the only outcome for an oyster is death, half-infective dose *K* was approximated by the half-lethal dose (LD_50_) of *V. aestuarianus* via contaminated-seawater exposure. LD_50_ was estimated using a dose–response model described by a four-parameter log-logistic function. This model was fitted to data from the individual triplicate trials, which were conducted at different doses (Table 2). A 95% confidence interval (CI) of LD_50_ was computed from the approximate standard errors calculated by the delta method (40). Because we assumed that a lower bacterial concentration would infect an oyster but would need more time to provoke oyster death than the usual duration to estimate the LD_50_, (7 days), a range of probable values was assigned to *K* by fitting a uniform distribution ranging between the bounds of the LD_50_ 95% CI, with the lower bound being lowered by one Log value.

**Table 2:** Observed final (12 dpe) number of surviving oysters in the laboratory transmission trials.

**Trial #**	**Exposure concentration of bacteria in water (dose W_**0**_)**	**Final number of surviving oysters in individual trials (*N* = 10 oysters)**	**Final number of surviving oysters in small population trials (*****N*** **= 3 populations of 10 oysters)**		
			**Triplicate #1**	**Triplicate #2**	**Triplicate #3**
1	Low = 4,140 bacteria/mL	10	NA^1^	NA^1^	NA^1^
	High = 41,400 bacteria/mL	4	NA^1^	NA^1^	NA^1^
2	Low = 29,200 bacteria/mL	8	8	6	6
	High = 73,900 bacteria/mL	2	8	7	3
3	Low = 3,410 bacteria/mL	8	7	7	10
	High = 8,120 bacteria/mL	6	6	6	5

1 *NA : non available*.

Exposure rate (*a*, per day) was estimated as a relative difference between the counts of bacteria before and after the 24 h of exposure to contaminated seawater. This daily exposure rate was assumed to be constant throughout the duration of the experiments. The latent period (1/ρ, days) was defined as the time from exposure to contaminated seawater to the start of bacteria shedding. The infectious period (1/*r*, days) was defined as the period from the start of oyster shedding to death. The bacteria shedding rate (*e*, bacteria/mL per oyster per day) was determined daily by measuring bacterial concentration in seawater. Free-living–bacteria lifetime in seawater (1/ξ, days) was defined as the number of days *V. aestuarianus* could be detected in the seawater.

Observed distributions of these parameters were described by the mean, mode, median, quartiles, minimum, and maximum. Independence of the parameters was assessed by calculating the Spearman correlation coefficient.

For each of the model parameters, the observed mode from experimental data was chosen as the most probable value. When the parameters were not independent, they were represented by the estimated mode, from distributions that were produced using an Approximated Bayesian Computation (ABC) approach (see below).

As initial conditions, the population size was modeled with 10 susceptible oysters to mimic the experiments in the 3 L tanks for 12 days. Seawater was supplemented with bacteria at different initial concentrations (W_0_), to reproduce the exposure to contaminated seawater in the 12 population experiments (Table 2).

#### Model Outputs

Five outputs were chosen to characterize the infection course. Two were dynamic over time: the number of live oysters (S+E+I) and the concentration of bacteria in water (W). Three were point outputs: the maximum prevalence (i.e., the maximum number of infected E and infectious I oysters during an infection simulation), the generation time, and the basic reproduction number R_0_.

The generation time was defined as the period between the onset of the infectious period in a primary case and the onset of the infectious period in a secondary case. It was defined from the sum of the average latent and infectious periods (41).

The basic reproduction number, R_0_, is defined as the average number of secondary infections caused by one infected entity (animal or free-living bacteria) introduced into a fully susceptible population (41), and it determines whether a disease will spread on average within a population. R_0_ carries information on the magnitude of the transmission during generation time. R_0_ was calculated using the next generation matrix approach (42, 43). In our system, the variables representing infectious states were E, I, and W. Progression from E to I was considered not a new infection but rather the progression of an infected oyster through disease stages. Contaminated water W was filled by shedding while new E animals occurred because of W>0. See Supplementary Material for more details on the calculations. Associated matrices are [equations (6) and (7)]:

(6) $$\begin{array}{lll} T & = & \left(\begin{array}{ccc} 0 & 0 & \frac{aKS_{0}}{{\left(K+W_{0}\right)}^{2}}\\ 0 & 0 & 0\\ 0 & e & 0 \end{array}\right) \end{array}$$

(7) $$\begin{array}{lll} \Sigma & = & \left(\begin{array}{ccc} -\rho & 0 & 0\\ \rho & -r & 0\\ 0 & 0 & -\xi \end{array}\right) \end{array}$$

(8) $$\begin{array}{lll} with-T.\Sigma^{-1} & = & \left(\begin{array}{ccc} 0 & 0 & \frac{aKS_{0}}{\xi {\left(K+W_{0}\right)}^{2}}\\ 0 & 0 & 0\\ -\frac{e}{r} & \frac{e}{r} & 0 \end{array}\right) \end{array}$$

where the transmission matrix *T* contains the terms related to new infections and the transition matrix Σ contains all the remaining terms, i.e., all exits from the infected classes and all entries into the infected classes for other reasons than generation of a new infected entity, such as different stages of infectiousness.

Basic reproduction number R_0_ is the dominant eigenvalue of matrix *-T* Σ ^−1^ [equation (9)]:

(9) $$\begin{array}{l} {\text{R}}_{0}=\sqrt{\frac{\text{eaK}{\text{S}}_{0}}{\text{r}\xi {\left(\text{K}+{\text{W}}_{0}\right)}^{2}}} \end{array}$$

The distribution, median, and 95% CI of the 10,000 simulations were examined for each of the model outputs.

#### Sensitivity Analysis

To identify the most influential parameters toward model outputs and to investigate interactions between parameters, a global variance-based sensitivity analysis was performed via the extended Fourier Amplitude Sensitivity Test (eFAST) method (44). The main order sensitivity index (direct effect) and the total order sensitivity index (including also interaction effects) were computed to describe the total influence of each parameter on model outputs. A parameter was considered to be a key parameter if it contributed to at least 20% of the variance of one of the model outputs.

Two aggregated outputs of the model were considered: the maximum prevalence and the basic reproduction number R_0_. Two additional dynamic outputs were considered: the number of surviving oysters and the bacterial concentration in seawater as functions of time.

The seven parameters (i.e., the epidemiological parameters and the initial exposure dose W_0_) varied simultaneously within their entire allowable range, with 10,000 scenarios for each parameter. Parameter ranges were defined by the 0.25 and 0.75 quantiles of observed experimental distributions for the six observable parameters, and by the bounds of LD_50_ 95% CI for the half-infective dose (*K*), lowering the lower bound by one Log value to account for the uncertainty of the use of the LD_50_ proxy.

This approach resulted in 70,000 scenarios. Because the model was stochastic, 500 repetitions were run, and outputs were averaged per scenario.

#### Parameter Estimation Using Approximate Bayesian Computation

Non-observable or non-independent epidemiological parameters were estimated using the number of surviving oysters during 12 dpe observed in the 12 replicate trials conducted on small populations of 10 oysters in tanks. Given that the likelihood evaluation of the model was not straightforward when only these data were employed, an Approximate Bayesian Computation (ABC) method was used. The ABC method consists of studying the similarity between observed and simulated data from intensive simulations, without the need for explicitly evaluating the likelihood function (45). The general principle consists of generating multiple parameter sets from prior distributions. For a sampled parameter set, a model simulation is performed. Then, summary statistics of this simulation are compared with the observed data using a metric and a tolerance. The parameter set is retained if the difference between the simulation and the observation does not exceed the tolerance. All the parameter sets thus retained are used to approximate the posterior distribution. To minimize the number of model simulations, and therefore computation time, the adaptive Monte Carlo ABC iterative algorithm (ABC-APMC) proposed by Lenormand et al. (46) was used. It consists of a sequential sampling with a sequence of decreasing tolerance levels leading to the approximation of the posterior distribution with an increasing quality. The proportion of best-fit simulations retained to update the tolerance threshold at each step was 0.2, and the stopping criterion (i.e., the proportion below which the algorithm stops the iterations and accepts the newly generated distributions) was 0.01.

The number of surviving oysters during 12 dpe observed in the 12 population replicate trials for the different experimental conditions at each time step were used as summary statistics. The Euclidian distance was used to compare simulations and observations. The values of the three observable parameters were fixed to the mode of observed values in the experiments. For non-independent parameters, bounds of the initial prior distribution were defined using the minimum and maximum observed values. For non-observable parameter *K*, the bounds of LD_50_ 95% CI were used, the lower bound being lowered by one Log value to account for the uncertainty of the use of the LD_50_ proxy. The parameter sets (or scenarios, *n* = 5,000) constituting the initial prior distributions were defined by a Latin hypercube sampling scheme across previously described parameter value ranges.

The posterior distributions of estimated parameters were summarized by the mode as the most probable value, and the 0.025 and 0.975 quantiles as the 95% credible interval.

#### Model Validation

The model was validated by comparing predicted values with the daily number of surviving oysters during 12 dpe and the bacterial concentration in seawater during 8 dpe which were observed in the 12 replicate trials conducted on small populations of 10 oysters in tanks. The average observed and simulated survival curves were compared by the logrank test (statistical significance threshold *p* < 0.05). The observed and simulated bacterial concentrations in seawater were compared by sight because of the small amount of observed data preventing statistical testing.

The compartmental model was solved in R software version 3.4.0 (47). The dose–response curve was fitted and LD_50_ was estimated using package *drc* version 3.0–1 (48), sensitivity analysis was performed using packages *mtk* version 1.0 (49) and *multisensi* version 2.1–1 (50), and ABC was performed using package *EasyABC* version 1.5 (51).

## Results

### Experimental Transmission Results

The values of epidemiological parameters were heterogeneous among individuals (Table 3, Supplementary Figure 1). In the experiments (Table 3), the average exposure rate to *V. aestuarianus* in seawater was 0.75, but this parameter highly varied among individual oysters. Once infected, an oyster became infectious 4 dpe on average, for only 1 day before dying of vibriosis, and daily shed ~1.9 × 10^5^ bacteria/mL. The bacterium *V. aestuarianus* remained in seawater for 2 days on average. At the end of the individual trials, six oysters out of 10 were still alive on average (Table 2). At the end of the experiment on small oyster populations, seven oysters out of 10 were still alive on average (95% CI: 4; 10; Table 2).

**Table 3:** Observed distribution characteristics of the epidemiological parameters in the individual laboratory experiments.

**Epidemiological parameters**	**N**	**Min**	**Q1**	**Median**	**Q3**	**Max**	**Mode**	**Mean**
Rate of exposure to contaminated water (*a*)	42	0.36	0.61	0.74	0.91	1	0.75	0.75
Bacteria shedding rate (*e*)	62	1,030	12,250	34,150	167,500	1,990,000	3,980	190,296
Latency period (1/ρ)	22	2	3.25	4	5	8	4.5	4.27
Infectious period (1/*r*)	22	0	1	1	2	3	1	1.18
Free-living bacteria lifetime in seawater (1/ξ)	54	1	1	2	3	3	1.5	1.94

*Units are the same as in Table 1*.

### Preliminary Parameter Calibration

**Figure 3:**
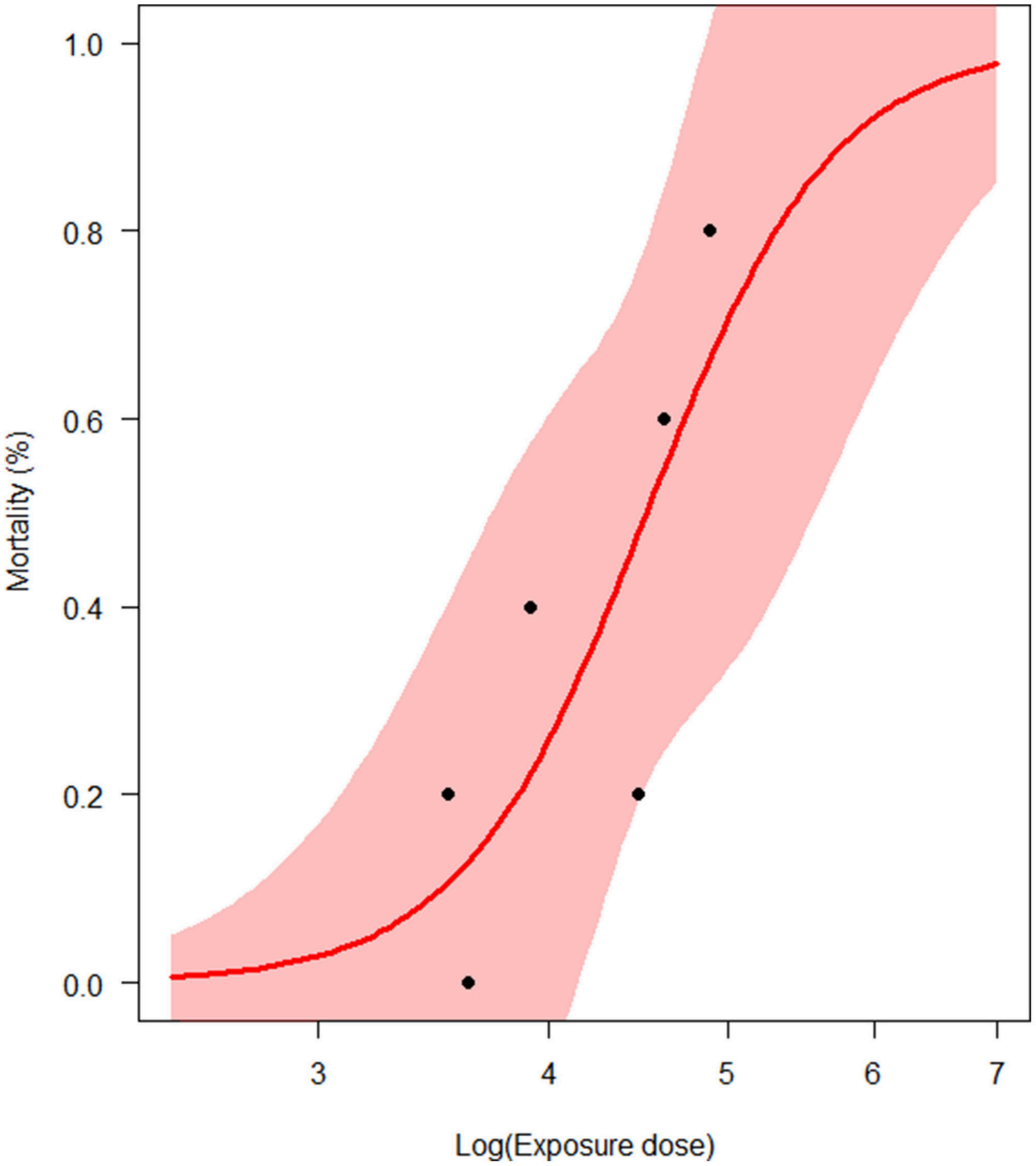
Estimation of the half-lethal dose (LD_50_) of *Vibrio aestuarianus* infection (*N* = 6 experimental trials). The pink area represents the 95% confidence interval of the estimates.

LD_50_ was estimated at 3.3 × 10^4^ bacteria/mL with the corresponding 95% CI (8.2 × 10^3^; 1.3 × 10^5^), i.e., 4.51 Log (bacteria/mL), 95% CI (3.91; 5.11; Figure 3). Thus, the concentration of bacteria in water that yields a 50% chance of catching the infection in 1 day (*K*) was first estimated as ranging from 8.2 × 10^2^ to 1.3 × 10^5^ bacteria/mL, i.e., from 2.91 to 5.11 Log(bacteria/mL). For each parameter, the observed mode was chosen as the most probable value of the parameter (Table 3). Except for a moderate positive association observed at a high bacterial dose between the bacteria shedding rate (*e*) and free-living bacteria lifetime in seawater (1/ξ), Spearman correlation coefficients were not significant for each pair of parameters (Table 4).

**Table 4:** The matrix of correlations between the epidemiological parameters (observed values; white cells: high bacterial dose, gray cells: low bacterial dose; Spearman coefficients: ^*^p < 0.001).

**Epidemiological parameter**	**a**	**e**	**1/ρ**	**1/r**	**1/ξ**
a		−0.079	0.339	−0.031	0.230
e	0.307		0.068	0.318	−0.051
1/ρ	−0.186	−0.147		−0.033	−0.194
1/*r*	−0.571	−0.190	0.078		0.059
1/ξ	0.012	0.510*	−0.186	0.082	

### Identifying Key Parameters

**Figure 4:**
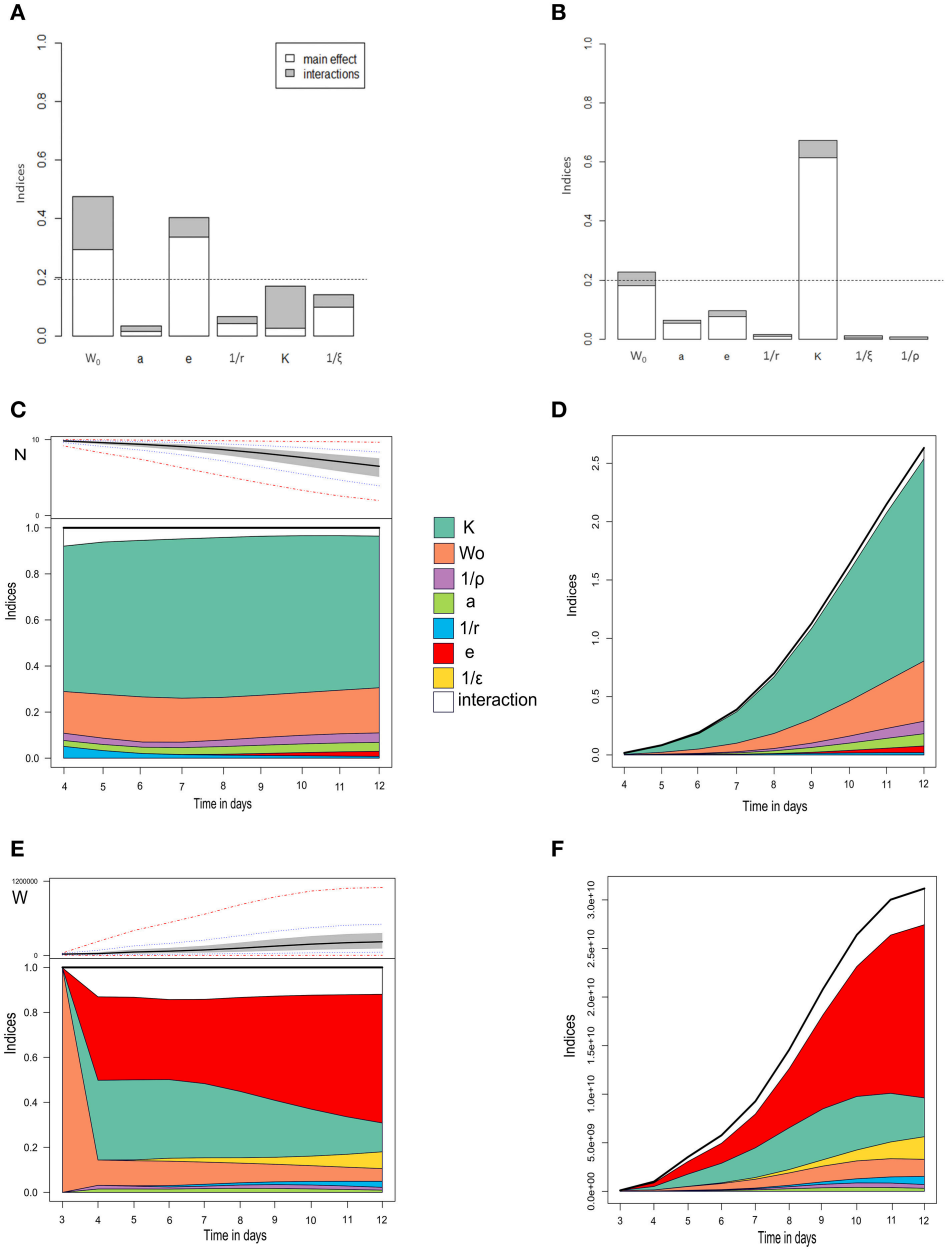
Global sensitivity analysis of three model outputs. **(A)** The basic reproduction number R_0_. **(B)** Maximum prevalence. **(A,B)** Main effects are in white, interactions in gray. The horizontal dotted line is the significance threshold (20% of variance). **(C,D)** Number of surviving oysters over time, with normalized **(C)** and non-normalized sensitivity indices **(D)**. **(E,F)** Bacterial concentration in seawater over time, with normalized **(E)** and non-normalized sensitivity indices **(F)**. In **(C)** and **(E)**, the upper subplots show the extreme (red dash-and-dot curves), percentiles (dotted blue curves), interquartile areas (gray), and median (bold curves) output values at every time point, while the lower subplots show sensitivity indices at every time point for main effects and interactions. W_0_, initial bacterial concentration; a,rate of exposure to seawater; e, bacteria shedding rate; 1/r, infectious period; K, half-infective dose; 1/ξ, free-living bacteria lifetime in seawater.

Figure 4 presents the main and total order sensitivity indices for the six parameters and the initial exposure dose on four of the selected model outputs. Three key parameters were identified: the half-infective dose (*K*), the bacteria shedding rate (*e*), and the initial exposure dose (W_0_). The basic reproduction number R_0_ was highly sensitive to the bacterial shedding rate (*e*, 41% of the R_0_ variation explained) and the initial exposure dose (W_0_, 48%; Figure 4A). The other parameters had a negligible effect (< 20%).

The maximum prevalence was highly sensitive to the half-infective dose (*K*), which explained 67% of its variation (Figure 4B), and, to a lesser extent, to the initial exposure dose (W_0_, 22%). The other parameters had a negligible effect (< 20%). The number of surviving oysters over time (Figures 4C,D) was highly sensitive to the half-infective dose (*K*, 70% of the variance explained) and, to a lesser extent, to the initial exposure dose (W_0_, 22%). The variability between simulations was low for the first 4 dpe, preventing any robust interpretation. The parameter influence was almost constant throughout the 12 days of the experiment (Figure 4C). Other parameters had a negligible effect (< 20%), and there were minor interaction effects. The concentration of bacteria in seawater was first highly sensitive to the initial exposure dose (W_0_), which influence rapidly decreased. After 4 dpe, bacteria concentration in seawater was highly sensitive to the bacteria shedding rate (*e*, 40–60%) and the half-infective dose (*K*, 13–26%; Figures 4E,F). The variability between simulations was low for the first 3 dpe, preventing any robust interpretation. After 8 dpe, bacteria concentration in seawater was mostly sensitive to the bacteria shedding rate. Other parameters had a negligible effect (< 20%).

### Improvement of Parameter Estimation by ABC

**Figure 5:**
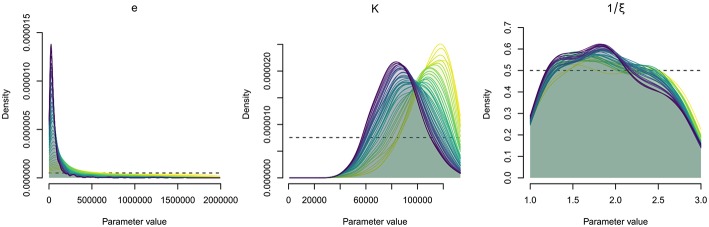
Parameter estimation by Approximate Bayesian Computation (ABC). Distributions of probability density of the epidemiological parameters, for sequential simulation steps (39 steps, 157,000 simulations); *e*, bacteria shedding rate; *K*, concentration of bacteria in water that yields a 50% chance of catching the infection; 1/ξ, free-living bacteria lifetime in seawater. The dotted lines show the prior distribution.

The posterior distributions (probability density) of the estimated parameter values are illustrated in Figure 5. The final estimated parameter values are given in Table 5. Estimations led to posterior distributions different from the prior distributions for the bacteria shedding rate (*e*) and the half-infective dose (*K*). The sharp posterior distribution obtained for the bacteria shedding rate highlighted that it is very likely that oysters shed relatively small quantities of bacteria. The concentration of bacteria in water that yields a 50% chance to get infected was estimated to be high with a large credibility interval. The posterior distribution for the free-living bacteria lifetime in seawater (1/ξ) was flat and rather similar to the initial prior distribution, preventing from any inference of its most probable value using available data.

**Table 5:** Estimated values and ranges of the model parameters of *Vibrio aestuarianus* transmission among oysters using Approximate Bayesian Computation (ABC; *N* = 1,000).

**Epidemiological parameter**	**Mode**	**95% credible interval**	
		**Lower bound**	**Upper bound**
Bacteria shedding rate (*e*)	72,684	6,690	374,535
Free-living bacteria lifetime in seawater (1/ξ)	1.88	1.04	2.91
Concentration of bacteria in water that yields 50% chance of catching infection in 1 day (*K*)	84,348	54,364	120,229

*Units are the same as in Table 1*.

### Simulating the Infection Dynamics

**Figure 6:**
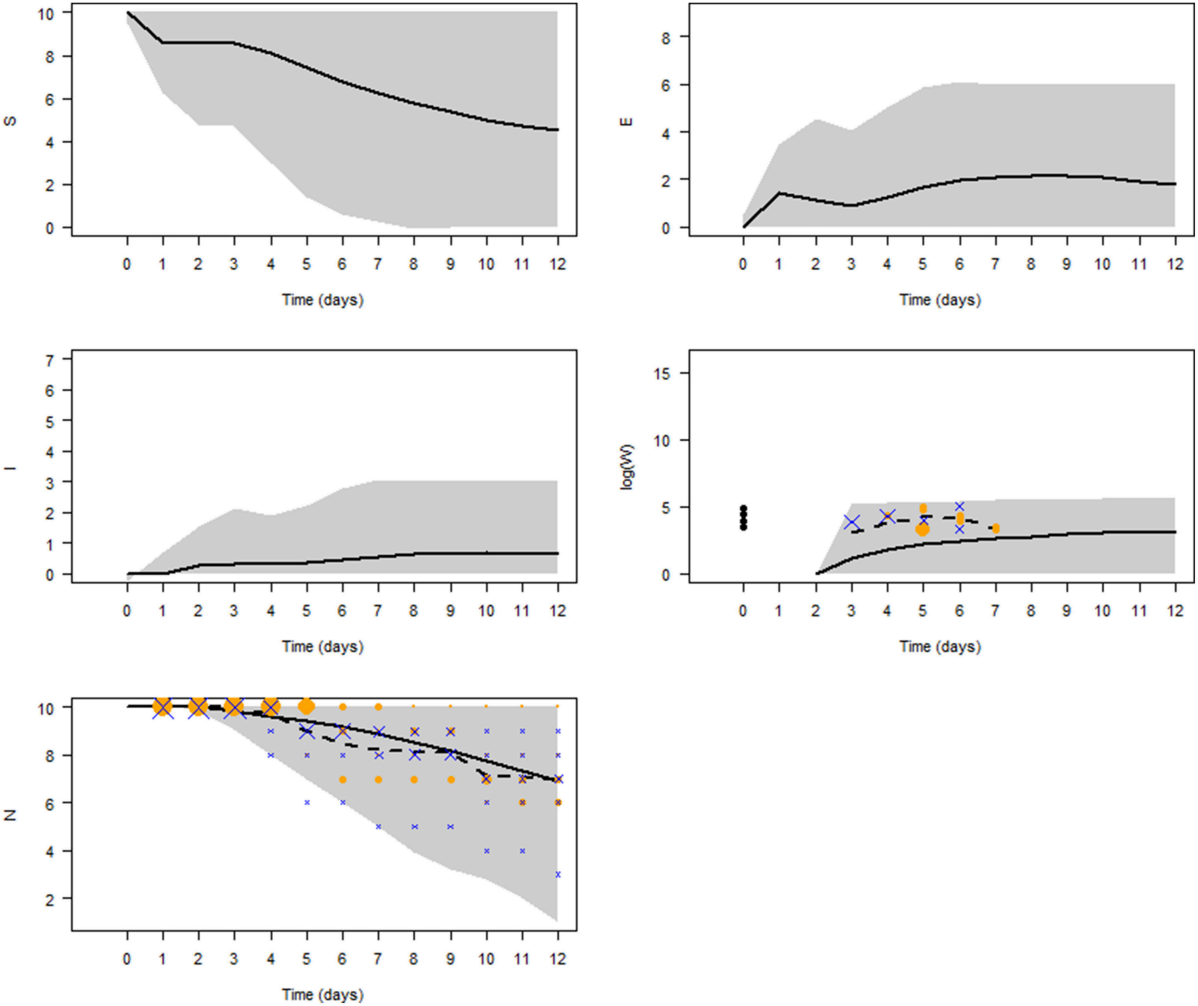
Model predictions. The five panels show the temporal dynamics of susceptible (S), exposed (E), infected (I) oysters, seawater bacterial concentration (W) in log scale, and total population size (N), using the epidemiological parameter values estimated by ABC. The black solid curves show mean values of 10,000 simulations. Gray shading represents the 95% credibility interval. Symbols denote the observed data in 3 L tanks during the laboratory experiments (orange dots: low doses; blue crosses: high doses), their size being proportional to the number of observations. The black dashed curve shows mean observed values in the experiments.

Figure 6 shows the simulated *V. aestuarianus* infection dynamics with the parameter values as estimated by ABC (Table 5). After 1 dpe of oysters to the contaminated seawater, vibriosis spread was observed in 61% of the simulations, with a median epidemic peak (maximum prevalence) of 60% (95% CI: 10; 80) reached at 9 dpe. The seawater concentration of *V. aestuarianus* slowly increased throughout the 12 days, reaching a median plateau at 2.5 × 10^5^ bacteria/mL (95% CI: 7.3 × 10^4^; 4.8 × 10^5^) at 10 dpe. At the end of the simulations, five oysters out of 10 were still alive on average (95% CI: 1; 9).

Figure 6 indicates that our model provided a good fit to the daily average number of surviving oysters observed in the 3 L tanks during the laboratory experiments (logrank test, p = 0.94), with the average number slightly overestimating the infection kinetics. Although the average model simulation results underestimated the bacterial concentration in seawater, the values observed in the experiments were always within the 95% CI of the model results.

Using the newly estimated parameter values, the basic reproduction number R_0_ was predicted to have a most probable value of 2.88 (95% CI: 1.86; 3.35). The generation time had a most probable value of 5.5 days. These data suggest a good transmission capacity of the bacteria, with an index case generating 2.88 secondary cases within 5.5 days on average once introduced in a population of susceptible oysters.

## Discussion

### Modeling Strategy

The current lack of knowledge on *V. aestuarianus* infection, notably on *V. aestuarianus* population dynamics, shaped our compartmental modeling strategy. Intensity models, where the pathogen population is explicitly modeled and the principal outcome is a measure of the number of pathogens, could have been employed if much information was available on the pathogen population, as is the case for *Perkinsus marinus* (27). We instead considered the epidemiological states of the host (*S, E*, and *I*) leading to simpler compartmental modeling. Notably, we adapted a model developed for *V. cholera* (29) because this aquatic bacterium also belongs to the genus *Vibrio*. This modeling strategy takes into account the rapid multiplication of the pathogen within the host and the short duration of the infection (14, 52). This approach is consistent with *V. aestuarianus* infection under experimental laboratory conditions (11, 12).

The assumptions underlying our model are a homogeneous contact process and a constant host population size (53). Mixing of oysters was assumed to be homogeneous within the 3 L tanks. Under the laboratory conditions, each oyster had an equal chance to come into contact with contaminated seawater, and the bacterial concentration in seawater was assumed to be homogeneous in the tank. Given the short period of the experiment, the closed-population assumption was fully acknowledged.

To better describe the transmission process in a small population, we used a stochastic model to incorporate the effect of chance, which may lead to small numbers of infectious oysters or transmission ceasing, and to produce a probability distribution of possible outcomes (54).

Our model is a simplified representation of a complex phenomenon according to the parsimony principle, especially as regards exposure to free-living bacteria. We assumed direct waterborne transmission of *V. aestuarianus* and added an explicit compartment representing the aquatic reservoir of bacteria in our model, as described for *V. cholera* (29). Only a small amount of field data on *V. aestuarianus* isolation from seawater, plankton, and sediment is available (10, 38, 39, 55), and detection protocols (8) did not allow discriminating virulent from non-virulent strains. Accordingly, the importance of plankton and sediment compartments in *V. aestuarianus* population dynamics for its transmission to oysters (i.e., reservoir, vector, intermediate hosts, and so forth) is still unknown. This situation prompted us to designate the free-living stages of the bacteria outside the oysters—whether in seawater, sediment, or plankton—as a single compartment. Moreover, we did not make any assumption about the detailed process of transmission of *V. aestuarianus* between oysters. We approximated the exposure rate (*a*) by the relative difference between the counts of bacteria before and after the 24-h exposure to contaminated seawater. This approach may be consistent with the filtration of infective particles released by infected individuals including a dose dependence and dilution via the volume of the water column hypothesized for suspension feeders (25, 56) such as oysters.

### Epidemiological Parameter Estimation

Combining the mathematical model with experimental data, we have been able to produce new knowledge about crucial epidemiological parameters. This coupled experiment–modeling approach enabled us to maximize the utilization of information (obtained through the experiments) in the model. We conducted a small number of experiments on small oyster populations owing to the logistical constraints of the numerous daily and individual measures. The initial exposure doses were difficult to accurately reproduce between the three experiments. This state of affairs may partly explain the observed heterogeneity in the parameters. Some parameters were directly observable and were measured in the experiments; others could not be observed directly or were not independent from each other. For the latter, we represented their uncertainty using uniform distributions between their minimum and maximum observed values in the experiments. We fed the model with parameter values determined, directly using the observed mode for the observable parameters or the distribution and estimated values for the others. The ABC method enabled reducing the uncertainty for two parameters out of the three, with variable accuracy.

We determined the most probable exposure rate at 75% per day, which is consistent with the small volume of contaminated seawater (300 mL) in our experimental settings thereby maximizing the exposure.

Our results uncovered the most probable latency period of 4.5 days and the most probable infectious period of 1 day. These findings are in agreement with previous experiments (12).

The bacteria shedding rate manifested high individual variability and was estimated to be between 6.7 × 10^3^ and 3.7 × 10^5^ bacteria/mL per oyster per day (95% credibility interval), with a most probable value estimated at 7.2 × 10^4^ bacteria/mL per oyster per day. Most of the oysters shed ~3.7 × 10^4^ bacteria/mL per oyster per day, but some individuals showed much greater values: up to 2.0 × 10^6^ bacteria/mL per oyster per day were observed in the experiments. Because several measures were found to have this order of magnitude in the experiments (Supplementary Figure 1), this heterogeneity did not come from measurement error but reflected realistic variations. Thus, some oysters may be referred to as supershedders of *V. aestuarianus*. Our estimate was slightly lower than the order of magnitude of shedding of 10^5^ bacteria/mL per oyster after 20 h reported in another work (12), where the studied parameters were assumed to be independent. Given that our results revealed a correlation between the bacteria shedding rate and free-living bacteria lifetime in seawater, our estimate was expected to be lower.

Our results showed only short free-living bacteria lifetime in seawater at 20°C, ranging from 1 to 3 days, but the available data prevented us from any inference of the most probable value for this parameter. This lack of estimation may be due to its relative lowest influence compared to the two other estimated parameters (bacteria shedding rate and half-infective dose) on the selected model outputs related to disease spread (i.e., R_0_, maximum prevalence, oyster survival curve, and bacteria concentration in seawater), as shown by the global sensitivity analysis. Nonetheless, this range is consistent with another study, which showed viability of *V. aestuarianus* in seawater after < 5 days at 25°C under differential experimental salinity conditions (39).

We estimated the half-lethal dose (LD_50_) at 3.3 × 10^4^ bacteria/mL (95% CI: 8.2 × 10^3^; 1.3 × 10^5^), which is lower by 1 Log than previous estimate (12), and the concentration of bacteria in water that yields a 50% chance of catching the infection (half-infective dose) ranged from 5.4 × 10^4^ to 1.2 × 10^5^ bacteria/mL, with a most probable value of 8.4 × 10^4^ bacteria/mL, i.e., 4.92 Log (bacteria/mL). This estimate is close to the minimal infective dose previously estimated to be ~4.0 × 10^4^ bacteria/mL (12). These close estimates of LD50, half-infective dose, and minimal infective dose strengthen the assumption that the oyster once infected never returns to a truly uninfected state, with death as the sole outcome.

### *V. aestuarianus* Transmission Insights

Our model provides important insights on transmission of *V. aestuarianus* at local scale, where the exposure to contaminated seawater is constant and homogeneous for every individual oyster in the population, and when a short period (12 days) is considered.

The two key factors that determine the spread of any infectious disease are the basic reproduction number, R_0_, and the generation time. First, R_0_ represents the number of new infections that arise on average from one infected oyster when the entire population is susceptible, i.e., at the onset of an epidemic. This is an approximate measure of infection transmissibility among animals within a population, and thus a key element for understanding infectious diseases. Our results show that an index case, i.e., an infected oyster or contaminated seawater with *V. aestuarianus*, can generate secondarily on average 2.88 (95% CI: 1.86; 3.35) new infected oysters. For oyster diseases, R_0_ has rarely been estimated so far, except for Dermo disease caused by the parasite *Perkinsus marinus*, for which estimation has yielded a large value, up to 90 (27). Second, generation time refers to the interval between the infection of an individual and the subsequent transmission to other individuals. We estimated the most probable value for generation time to be 5.5 days under our laboratory conditions. As a first conclusion, *V. aestuarianus* shows a good transmission efficiency in oyster populations under experimental conditions: it combines moderate R_0_ and quite short generation time, with each index case producing ~2.88 secondary cases within roughly 1 week. The expected lifespan of infected oysters is short (less than a week), and they are infectious for only one day, shedding daily an amount of bacteria into the seawater, 4.86 Log(bacteria/mL), that is almost equivalent to the half-infective dose [4.92 Log(bacteria/mL)] on average, but which can be also highly variable (2 Log range) within a homogeneous oyster population. The bacterium has short free-living lifetime outside the oyster in seawater, < 3 days. Thus, *V. aestuarianus* should be able to persist mainly in populations of oysters of high local density with close interactions.

The parameters mostly influencing variations in the basic reproduction number, R_0_, and identified by a global sensitivity analysis suggest a dose-dependent virus transmission: the higher the bacteria shedding rate, the higher the initial exposure dose or the lower the half-infective dose, the larger is the number of new infections arising from one infected oyster in a fully susceptible population. The dose-dependent mortality induced by *V. aestuarianus* has been previously examined experimentally (9, 12). The half-infective dose was identified as a key parameter, i.e., the factor explaining most of the variation in most of the selected model outputs (maximum prevalence, oyster survival curve, bacterial concentration in seawater over time). These results are consistent with a local waterborne transmission mode (16) and the filtration-based models (25), in which the more infected and shedding oysters there are, the higher the local concentration of bacteria is likely to be, for given decay and dispersal rates. Hence, a threshold concentration of bacteria may be required to induce infection, meaning that a sufficient number of susceptible oysters is needed to support infection spread, but also that a sufficient number of infectious oysters (16) or the presence of supershedder oysters are needed to obtain a high initial exposure dose and to start an epidemic. The prevalence of infection in the population thus does not dependent on oyster density at a local scale. The shedding rate was distributed over a large range of values, showing a high heterogeneity (Supplementary Figure 1), meaning that even if the oyster density is low, a few supershedders may strongly contribute to the infection spread and magnitude. This transmission mode is consistent with the ecology of oyster beds or oyster farms, which are aggregated populations with close interactions and large local numbers of individuals.

Prediction robustness can be further improved by better estimating two key parameters influencing *V. aestuarianus* spread: the half-infective dose and the bacteria shedding rate, as identified by the global sensitivity analyses. Hence, further experimental effort should be best directed to reduce these parameter uncertainties (57), which will in turn improve our understanding of the infection spread.

### Study Limitations

In our study, small-scale transmission experiments were designed to ensure the best practices (58). Transmission experiments under controlled conditions have an advantage over a field study: an infection chain can be deliberately started (58). When studying natural infections, the use of contact animals, rather than artificially inoculated ones with presumed higher infectiousness, has been recommended to start the studied infection chain (58). Nonetheless, we started the experimental infection and initiated the modeled infection by means of contaminated seawater (balneation infection protocol). We preferred such a balneation infection protocol to a cohabitation one because we could standardize the bacterial concentration in the seawater more easily, given the high variability of the shedding rate of infectious oysters. Thus, our experimentally initiated infections in small populations of 10 oysters in the tanks possibly did not strictly represent a transmission process. All the 10 oysters were exposed together for 24 h in a tank with titrated contaminated seawater, then were transferred to clean water for the 12-day monitoring. Hence, we cannot be sure that what we observed in tanks was transmission, a point-source outbreak without any transmission, or a mixture of both, as revealed in the experimentally initiated epidemics of infectious pancreatic necrosis in rainbow trout fry (59). This may explain the overestimation of the kinetics of infection by the model involving the baseline range of parameters sampled from the distributions fitted to experimental individual data. Accordingly, our results may underestimate the transmissibility of vibriosis. To untangle this issue, further experiments should include a single contact oyster exposed to contaminated seawater for 24 h in a tank, then transferred into clean water with 10 naïve oysters for 12 days of monitoring. The shedding rate of the contact oyster should be carefully estimated beforehand.

Transmission experiments under controlled conditions have a disadvantage that result extrapolation may be difficult (58), particularly for the key process of transmission (30). The mode of transmission determines the probable response for a disease to control actions (19) and may change with the study scale, i.e., inter- or intrapopulation (16, 19). Thus, it is crucial to know how transmission scales with population size and/or density (19).

### Concluding Summary and Perspectives

Here, our model showed that the bacteria shedding rate, the half-infective dose and the initial bacterial exposure dose have a major influence on the outputs related to the extent of infection by *V. aestuarianus* at a small population scale. To control the transmission of bacteria to a susceptible oyster population at such a local scale, it is therefore necessary to identify the processes that increase or decrease oyster exposure to contaminated seawater. These processes may be the factors related to the oyster itself, such as filtration rate (26), sensitivity to the infection (11) or bacteria shedding, or to environmental stressors that increase bacteria shedding by infectious oysters or sensitivity of susceptible oysters to infection (60), the duration of exposure to contaminated seawater (i.e., water renewal or confinement), or hydrodynamics that may drive particle loss or diffusion-like processes in the water column (26). Free-living bacteria can be transported across long distances by water currents, leading to transmission of the infection between oyster populations. At a small scale, all free-living bacteria rain down equally regardless of their source on all susceptible oysters. At a larger scale, however, the patchy distribution of oysters and neighborhood characteristics lead to a heterogeneous exposure: free-living bacteria are dispersed only locally but the rain of particles is nonetheless homogeneous (61) because of concentration gradients of free-living bacteria owing to the dilution effect.

Our model mimics the *V. aestuarianus* infection in an oyster population within a controlled environment under laboratory conditions. This is a first step toward understanding the epidemiology of this infection in the field. Indeed, physical variation of the environment is crucial for marine diseases, especially in marine ectothermic invertebrates such as mollusks (62). In the field, mollusk mortality cases associated with *V. aestuarianus* detection mainly occur in summer (1, 3). In a mesocosm study conducted in winter, infected oysters could survive when the sea temperature was low (~5°C) and the infection could be revealed in the laboratory by a thermal stress assay (10). Therefore, transmissibility of the infection over time may be modulated (increased or decreased) by environmental factors. Further studies are needed to assess the effect of seawater temperature and seasonality on the disease kinetics, as estimated for *Perkinsus marinus* (27), and especially on epidemiological parameters.

Because the mode of transmission may vary according to the spatial scale, the choice of an appropriate mathematical model may also depend on the spatial scale of interest (16, 19). For disease management purposes, the management unit needs to be well defined to describe transmission adequately because this element determines the probable response of the disease to control actions (19). Future research should consider embedding this local scale disease model within an oyster metapopulation landscape (21, 23) and hydrodynamics (63) to develop a marine epidemiological simulation model for evaluating the effectiveness of various control strategies against *V. aestuarianus* infection.

## Ethics Statement

All the methods were carried out in accordance with guidelines. Pacific oyster is not an endangered or protected species, and not vertebrate. The oysters used in this study were farmed.

## Author Contributions

M-AT and DT conceived and conducted the experiments. CL and PE developed the model structure and coding. CL, GB, and PE performed the modeling and data analyses. CL designed the numerical and statistical analyses. M-AT, CB, CL and GB contributed to figures. CL, M-AT, DT, GB, and PE discussed the results and contributed to the writing of the manuscript. All the coauthors reviewed the manuscript and approved the final version for publication.

### Conflict of Interest Statement

The authors declare that the research was conducted in the absence of any commercial or financial relationships that could be construed as a potential conflict of interest.
