# Supplementary material for: Modeling the Transmission of Vibrio aestuarianus in Pacific Oysters Using Experimental Infection Data
Source: Front Vet Sci. 2019 May 14;6:142. doi: 10.3389/fvets.2019.00142 (PMC6527844; doi:10.3389/fvets.2019.00142)
Supplement: Supplementary file 1 [file Data_Sheet_1.docx]

Supplementary Material

**Modeling the transmission of *Vibrio aestuarianus* in Pacific oysters~~,~~ using experimental infection data**

Coralie Lupo^*^, Marie-Agnès Travers, Delphine Tourbiez, Clément Félix Barthélémy, Gaël Beaunée, Pauline Ezanno

*** Correspondence:** Corresponding Author: [clupo@ifremer.fr](mailto:clupo@ifremer.fr)

The Next Generation Matrix (NGM) method has been applied ([1](#_ENREF_1)) to derive the theoretical Ro calculation.

The ordinary differential equations (ODE) are:

$\frac{\mathrm{dS}}{dt} = -a\lambda\left( W \right)S$ (1)

$\frac{dE}{dt}= a\lambda\left( W \right)$S − $\rho E$ (2)

$\frac{dI}{dt}= \rho E-rI$ (3)

$\frac{dW}{dt}= eI- \xi W$ (4)

with:

$\lambda\left( W \right)= \frac{W}{K+W}$ , (5)

With N = S + E + I. This system has 3 infected classes, exposed oysters (E), infected oysters (I) and free-living bacteria in the seawater (W).

At the disease-free equilibrium (DFE), the disease has disappeared from the system or has not yet entered it, *i.e.* there is no infected or infectious hosts (E=0 and I=0) and there is no free-living bacteria in the seawater (W=0). The number of oysters is S=S_0_.

The matrix **T** corresponds to transmissions if E_0_=1 or I_0_=1 or if W_0_ bacteria are introduced into the system at the DFE. It includes all epidemiological events that lead to new infections. The matrix **Σ** corresponds to transitions among states, and includes all other events, *i.e*. all exits from the infected classes and all entries into the infected classes for other reasons than the generation of a new infected entity (infected animal of free-living bacteria).

In our system, we have 3 infected classes thus the matrices **T** and **Σ** are three-dimensional [equations (6) and (7)]. They are obtained from equations (2), (3) and (4) by separating the transmission events from other events. In the matrices, the upper left term corresponds to the partial derivative of the differential equation dE/dt with respect to E, considering I and W constant. The upper middle term corresponds to the partial derivative of the differential equation dE/dt with respect to I, considering E and W constant, and so on for other terms of the matrix. Especially, the only new infected entities generated are either shed bacteria (equal to *e* if one infectious animal is introduced at DFE), or newly infected animals (of state E, first line third column of matrix **T**) if seawater is initially contaminated at level W_0_.

$\mathbf{T}= \left( \begin{matrix} 0 & 0 & \frac{a{KS}_{0}}{(K+W_{0})^{2}} \\ 0 & 0 & 0 \\ 0 & e & 0 \end{matrix} \right)$ (6)

$\boldsymbol{\Sigma}= \left( \begin{matrix} -\rho& 0 & 0 \\ \rho& -r & 0 \\ 0 & 0 & -\xi\end{matrix} \right)$ (7)

The NGM is defined as **K** = –**T.Σ**^-1^

To find the inverse of a three-dimensional matrix $A= \left( \begin{matrix} a & b & c \\ d & e & f \\ g & h & i \end{matrix} \right)$, first we find the determinant (det) of matrix A:

det(A)=$aei+dhc+gbf-ahf-dbi-gec$

Second, we find the transpose of the cofactor matrix C:

C = $\left( \begin{matrix} +\left| \begin{matrix} e & f \\ h & i \end{matrix} \right| & -\left| \begin{matrix} d & f \\ g & i \end{matrix} \right| & +\left| \begin{matrix} d & e \\ g & h \end{matrix} \right| \\ -\left| \begin{matrix} b & c \\ h & i \end{matrix} \right| & +\left| \begin{matrix} a & c \\ g & i \end{matrix} \right| & -\left| \begin{matrix} a & b \\ g & h \end{matrix} \right| \\ +\left| \begin{matrix} b & c \\ e & f \end{matrix} \right| & -\left| \begin{matrix} a & c \\ d & f \end{matrix} \right| & +\left| \begin{matrix} a & b \\ d & e \end{matrix} \right| \end{matrix} \right)$ = $\left( \begin{matrix} ei-hf & fg-di & dh-ge \\ ch-bi & ai-gc & gb-ah \\ bf-ec & dc-af & ae-db \end{matrix} \right)$

And C^T^ = $\left( \begin{matrix} ei-hf & ch-bi & bf-ec \\ fg-di & ai-gc & dc-af \\ dh-ge & gb-ah & ae-db \end{matrix} \right)$

A^-1^ = $\frac{1}{det(A)}\times$ C^T^

We have: det(**Σ**) **=** $\left( -\rho\times-r\times-\xi\right)+\left( \rho\times0\times0 \right)+\left( 0\times0\times0 \right)-\left( -\rho\times0\times0 \right)-\left( \rho\times0\times-\xi\right)-\left( 0\times-r\times0 \right)= -\rho r\xi$

The determinant of matrix **Σ** is not null, thus **Σ** can be inversed.

Then **Σ** ^-1^ = $\frac{1}{-\rho r\xi}\times\left( \begin{matrix} r\xi& 0 & 0 \\ \rho\xi& \rho\xi& 0 \\ 0 & 0 & \rho r \end{matrix} \right)$ = $\left( \begin{matrix} -\frac{1}{\rho} & 0 & 0 \\ -\frac{1}{r} & -\frac{1}{r} & 0 \\ 0 & 0 & -\frac{1}{\xi} \end{matrix} \right)$ (8)

Thus **K** = –**T.Σ**^-1^ = $\left( \begin{matrix} 0 & 0 & \frac{a{KS}_{0}}{(K+W_{0})^{2}} \\ 0 & 0 & 0 \\ 0 & e & 0 \end{matrix} \right) \times\left( \begin{matrix} \frac{1}{\rho} & 0 & 0 \\ \frac{1}{r} & \frac{1}{r} & 0 \\ 0 & 0 & \frac{1}{\xi} \end{matrix} \right)= \left( \begin{matrix} 0 & 0 & \frac{a{KS}_{0}}{\xi(K+W_{0})^{2}} \\ 0 & 0 & 0 \\ \frac{e}{r} & \frac{e}{r} & 0 \end{matrix} \right)$ (9)

Then, to find the eigenvalues of **K**, let solve det(–**T.Σ**^-1^ - λI) = 0, I being the identity matrix.

B = –**T.Σ**^-1^ – λI = $\left( \begin{matrix} 0 & 0 & \frac{a{KS}_{0}}{\xi(K+W_{0})^{2}} \\ 0 & 0 & 0 \\ \frac{e}{r} & \frac{e}{r} & 0 \end{matrix} \right)-\left( \begin{matrix} \lambda& 0 & 0 \\ 0 & \lambda& 0 \\ 0 & 0 & \lambda\end{matrix} \right)= \left( \begin{matrix} -\lambda& 0 & \frac{a{KS}_{0}}{\xi(K+W_{0})^{2}} \\ 0 & -\lambda& 0 \\ \frac{e}{r} & \frac{e}{r} & -\lambda\end{matrix} \right)$

Det(B) = $-\lambda^{3}+\frac{e\lambda a{KS}_{0}}{r\xi(K+W_{0})^{2}}$

Det(B) = 0 $\underset{\Leftrightarrow}{}{-\lambda}^{3}+ \frac{e\lambda a{KS}_{0}}{r\xi\left( K+W_{0} \right)^{2}}=0\underset{\Leftrightarrow}{}\lambda=0 or \lambda^{2}= \frac{ea{KS}_{0}}{r\xi\left( K+W_{0} \right)^{2}}$

The largest eigenvalue corresponds to R_0_.

R_0_ = $\sqrt{\frac{ea{KS}_{0}}{r\xi\left( K+W_{0} \right)^{2}}}$

**Supplementary Figure 1.** Distributions of the epidemiological parameters as observed in experimental conditions (grey histogram) and most probable values used in the model: observed mode (blue vertical line) and value estimated by ABC (blue shading).


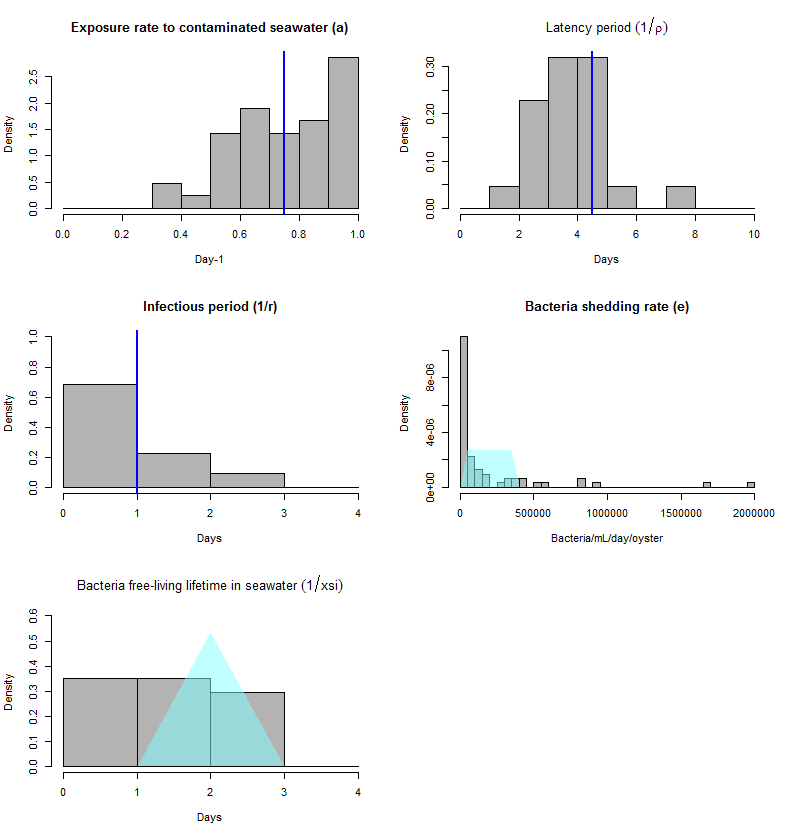


**Reference:**

1. Diekmann O, Heesterbeek JA, Roberts MG. The construction of next-generation matrices for compartmental epidemic models. *Journal of the Royal Society, Interface / the Royal Society* (2010) 7(47):873-85. Epub 2009/11/07. doi: 10.1098/rsif.2009.0386.
